# Narrative exposure therapy for immigrant children traumatized by war: study protocol for a randomized controlled trial of effectiveness and mechanisms of change

**DOI:** 10.1186/s12888-015-0520-z

**Published:** 2015-06-17

**Authors:** Samuli Kangaslampi, Ferdinand Garoff, Kirsi Peltonen

**Affiliations:** 1 School of Social Sciences and Humanities/Psychology, FI-33014, University of Tampere, Tampere, Finland; 2 Institute of Behavioural Sciences/Psychology, FI-00014, University of Helsinki, Helsinki, Finland

**Keywords:** NET, CBT, RCT, Trauma, Memory, Cognition, Resilience, War, Posttraumatic stress disorder, Children

## Abstract

**Background:**

Millions of children worldwide suffer from posttraumatic stress disorder (PTSD) symptoms and other mental health problems due to repeated exposure to war or organized violence. Forms of cognitive-behavioral therapy (CBT) are the most commonly used treatment for PTSD and appear to be effective for children as well, but little is known about the mechanisms of change through which they achieve their effectiveness. Here we present the study protocol of a randomized controlled trial (RCT) studying the effectiveness and mechanisms of change of Narrative Exposure Therapy (NET), a CBT-based, manualized, short-term intervention for PTSD symptoms resulting from repeated traumatization, in immigrant children traumatized by war.

**Methods/Design:**

We are conducting a multicentre, pragmatic RCT in a usual care setting. Up to 80 9–17-year-old immigrant children who have experienced war and suffer from PTSD symptoms will be randomized into intervention (NET) and control (treatment as usual, TAU) groups of equal sizes. The effectiveness of NET treatment will be compared to both a waiting list and the parallel TAU positive control group, on the primary outcomes of PTSD and depressive symptoms, psychological distress, resilience, and level of cognitive performance. The effects of the intervention on traumatic memories and posttraumatic cognitions will be studied as potential mechanisms of change mediating overall treatment effectiveness. The possible moderating effects of peritraumatic dissociation, level of cognitive performance, and gender on treatment effectiveness will also be considered. We hypothesize that NET will be more effective than a waitlist condition or TAU in reducing PTSD and other symptoms and improving resilience, and that these effects will be mediated by changes in traumatic memories and posttraumatic cognitions.

**Discussion:**

The results of this trial will provide evidence for the effectiveness of NET in treating trauma-related symptoms in immigrant children affected by war. The trial will also generate insights into the complex relationships between PTSD, memory functions, posttraumatic cognitions and cognitive performance in children, and help guide the future development and implementation of therapeutic interventions for PTSD in children.

**Trial registration:**

ClinicalTrials.gov NCT02425280. Registered 15 April 2015.

## Background

In 2014, 230 million children lived in countries and areas affected by armed conflicts [1]. With increasing emigration and refugeeism, millions of children who have experienced war now also inhabit countries with no active conflicts. Research confirms high rates of mental health problems among war-affected children [2], even higher than those of similarly affected adults [3]. Studies demonstrate both general dose-effect relationships between exposure to war and trauma-related stress symptoms and specific types of war experiences that are particularly traumatizing [2]. As many as 47 % of children exposed to war may suffer from posttraumatic stress disorder (PTSD) and 43 % from depression, while reviews and surveys suggest a prevalence of 10–30 % for PTSD among all refugee and asylum seeking children resettled into high-income countries [4, 5].

Though most trauma survivors recover with time, for some, posttraumatic stress symptoms due to war experiences in childhood show persistence for years and even decades [6, 7], and have been shown to be related to a number of psychological and physical health issues even in older age [8]. PTSD in children also appears to be connected to lower verbal memory function and overall cognitive performance [9, 10] and is linked to impairment in academic performance [11, 12]. In addition to the decreased quality of life and increased suffering of trauma survivors, PTSD carries enormous economic costs associated with loss of personal income, inability to work, as well as increased utilization of treatment and support services. For example, in 2004, the social and welfare costs of claims for incapacitation and severe disablement in the UK from severe stress reactions and PTSD amounted to £104 million per annum [13]. Posttraumatic stress symptoms in children due to organized violence are thus a pressing, global problem. Developing effective, evidence-based interventions to address this problem should be a public health priority globally.

In this paper, we describe a randomized controlled trial (RCT) evaluating the effectiveness and potential mechanisms of change of a targeted intervention, Narrative Exposure Therapy, NET [14], for children who suffer from posttraumatic stress symptoms due to experiences of war or armed conflict. To our knowledge, it is the first effectiveness study of NET as implemented in the context of the existing healthcare system of a high-income country. In this context, it is also the first trial to explore whether changes in the quality of traumatic memories and posttraumatic cognitions are potential candidates for effective mechanisms of change in PTSD symptom reduction among war-affected children in treatment. This study protocol aims to present all relevant information pertaining to this randomized controlled trial, as set out by the CONSORT 2010 guidelines [15].

For traumatized children in general, cognitive-behavioral therapy (CBT) has been repeatedly found to be effective in reducing PTSD and other symptoms [16–18], and trauma-focused cognitive-behavioral therapy (TF-CBT) is recommended as the primary treatment for PTSD for both adults and children in several countries [19,20]. For children affected by war specifically, a variety of group and individual interventions have been implemented and studied all over the world. Reviews have reported evidence on the effectiveness of such psychosocial interventions in alleviating PTSD, depression and anxiety symptoms in children affected by armed conflict [21–23]. However, reductions in symptoms have often been modest, and study designs have lacked rigor, with only a small minority representing RCTs.

The majority of intervention techniques among children traumatized by war are also based on CBT and its derivatives. CBT-based interventions for traumatic stress share similar creative, narrative, and cognitive elements, such as creative-expressive exercises (dream work and fantasy), cognitive restructuring, attention control, body-oriented methods, building a sense of safety, and providing psychoeducation. However, it is still largely unclear which specific treatment elements in these interventions might be most significant for recovery. Furthermore, there is a general lack of rationales based on clear theoretical frameworks as to which particular CBT tools are chosen for use in treatment [22]. Therefore, there have been calls for research on the underlying mechanisms of change that contribute to the success or otherwise of interventions among war-affected children, i.e., particular processes mediating their effectiveness [21].

### NET as a CBT-based intervention

Narrative Exposure Therapy is a manualized, individual, short-term intervention program for the treatment of PTSD resulting from exposure to organized violence or other repeated traumatic events. NET is based on CBT principles, with its development influenced by exposure-based and testimonial therapies [14]. The specific focus in NET is on habituation to and contextual anchoring of traumatic memories. This focus stems from the clinical model of repeated traumatization underlying NET, drawing on 1) dual representation theories of PTSD [24, 25], see also [26], and 2) Emotional Processing Theory and the idea of fear networks [27].

According to dual representation theory, during a highly emotional event, two differing types of parallel memory representations are encoded. The sensory, cognitive-emotional and physiological features of the event are stored in long-term perceptual memory [26], and have been called hot memories [14, 24], Situationally Accessible Memories [25] or sensation-near representations [28], while the contextual, verbalizable elements of the situation are encoded into episodic memory [26], and have been called cold memories [14, 24], Verbally Accessible Memories [25] or contextualized representations [28].

Based on Emotional Processing Theory, hot memories are thought to be stored as sensory-perceptual representational networks, containing memories of stimuli in different modalities, together with cognitive and emotional states experienced during the event. Such representational networks may be created for any emotionally significant event. However, in the case of a traumatic event, the generated representation (called a fear network) would be unusually expansive and contain a great number of sensory, cognitive, emotional and physiological elements, most of which were previously considered safe and non-threatening. The large number of such elements and the strong connections formed between them mean that activation of just one element in the network may be enough to activate the entire structure. As the activation of the network (such as, in the form of a flashback) is a frightening event, this leads to trauma survivors avoiding any elements included in the network, as well as any possible cues reminding them of these elements. This understanding of the disconnected encoding of sensory-perceptual memories accounts for the avoidance, intrusion and numbing (avoiding even positive emotional experiences) symptoms seen in PTSD.

Cold memories, for their part, are seen to be selective representations of the contextual and factual elements of the event, consciously and verbally accessible to the trauma survivor. For a single event, a fear network usually remains mostly connected to its cold memory counterpart, and some autobiographical context is maintained. However, if a new traumatic experience becomes integrated into an already existing fear network of previous traumatic experiences, this connection may be partly or wholly lost. Thus, with each additional traumatic event, the fear network grows, and the hot memories become increasingly disconnected from the contextual referents of the cold memories (such as time and place). Hence, with repeated traumatization, the resulting fear/trauma network may end up containing sensory elements and disconnected perceptual or physiological memories from many traumatic events, all mixed together with little spatio-temporal context.

Based on this clinical model of PTSD, the central treatment element in NET is activating sensory-perceptual representations of traumatic events, especially the most intensely emotional and most autobiographically fragmented ones, and reconnecting them to the contextual episodic memories of the events in question. In other words, NET aims to provide a distinct time and place for the disorganized, highly emotional memories trauma survivors have of their experiences.

As most survivors of organized violence have experienced a number of traumatic events, it is often difficult or impossible for them to identify a single worst event to be processed by traditional pure exposure methods. To address this difficulty, NET treatment begins by the participant constructing a representation of his whole life from early childhood up to the present, placing important events, both positive and traumatic, on a lifeline. All traumatic events identified in this manner are then narrated in chronological order. The trauma survivor is thus exposed to repeated and detailed elaboration of what happened during the traumatic events and may become desensitized to trauma reminders. Subsequently, physical and psychological hyperarousal and the need to avoid painful reminders should decrease. At the same time, survivors are assisted in integrating their conscious, verbally accessible memories and thoughts with sensory-based traumatic memories into coherent, emotionally versatile and meaningful stories of these important moments in their lives. Creating this trauma narrative and processing the trauma emotionally and cognitively contributes to the integration of the trauma and its meaning into an optimal self-concept and life history [14] and to the disconfirmation of maladaptive beliefs or appraisals that may have developed after the trauma.

### Previous research on NET

A review of five RCTs conducted on NET with adults concluded that good evidence already exists to support the use of NET in the treatment of PTSD among adult survivors of organized violence [29]. Promising evidence from three randomized trials is already available with children as well [5, 30, 31], reviewed in [29]. For refugee children in a high-income country (Germany), NET lead to reductions in symptom severity in all PTSD symptom clusters at 4 weeks post-treatment, sustained for 12 months, while there were no significant changes in a waiting list condition [5]. Among former child soldiers in Uganda, some of whom were adolescents and some young adults, NET reduced PTSD symptoms significantly more than either a program of academic catch-up and supportive counselling or a waiting list condition, with significant changes in PTSD symptom scores in 20 out of 25 participants in the NET group [31]. Further, Catani et al. found NET treatment to be at least as effective as a meditation-relaxation protocol in reducing PTSD symptoms and functional impairment in 8–14-year-old Sri Lankan children affected by both war and a very recent mass disaster (tsunami), though comparison to spontaneous recovery was not possible in these conditions [30].

### Current trial

The general objective of this study is to contribute to the search for the most effective, evidence-based intervention methods to help children traumatized by war. The study aims to achieve this objective by comparing the effectiveness of NET in the treatment of war-affected children suffering from posttraumatic stress reactions with both a waiting list condition and a treatment as usual (TAU) control condition in a parallel-group randomized controlled trial. In addition, the potential mediating and moderating effects of a number of factors related to memory and other cognitive processes will be explored. The trial is pragmatic by nature, being carried out inside the Finnish healthcare system and emphasizing direct applicability of its results to that system and others similar to it.

This trial aims to extend the evidence from previous RCTs [5, 30, 31] on the effects of NET on children’s psychopathology in at least six different ways.

First, in comparison to earlier studies carried out on NET with children, this trial benefits from a comparatively large and inclusive sample drawn from immigrant children settled or seeking to settle in Finland. Only one study has previously studied the use of NET with refugee or immigrant children in their new home country, with no active control group [5].

Second, all previous studies on NET with children up to 2014 have involved one or more of the developers of NET in some position. This trial will be carried out by independent researchers with no affiliation to the institutions involved in the development of NET.

Third, whereas much of the earlier research on NET may be more accurately described as efficacy studies, this trial is one concerned with effectiveness in a current real-world clinical framework. Thus, it tends towards the pragmatic end of the pragmatic-explanatory continuum, in the sense defined by the extension of the CONSORT statement on pragmatic trials [32]. It is to our knowledge the first such pragmatic effectiveness study on NET with children carried out in a high-income setting. The setting in question is the existing Finnish healthcare system, mostly in the Tampere region, including three outpatient clinics and two in-patient psychiatric wards. The clinicians carrying out the intervention and acting as assessors are healthcare professionals who would in any case treat these children, mostly psychologists, psychiatrists and psychiatric nurses, some with formal psychotherapy training. The pragmatic nature of the trial adds to its practical significance, as a new treatment method and targeted intervention will be rolled out in the context of the Finnish healthcare system.

Fourth, going beyond studying the simple effectiveness of NET, this trial aims at improving our understanding of the process of healing and recovery from posttraumatic stress symptoms due to war exposure. The trial does this by analysing some of the mechanisms of change that the theory underlying NET suggests would contribute to its success or otherwise.

Based on that theory, we expect that the effects NET has on posttraumatic stress symptoms would occur through recontextualizing disconnected sensory-perceptual memories, and linking them with their verbalizable episodic memory counterparts. This process would result in and be evinced by less fragmented memories of traumatic events with more spatial and temporal contextualization and coherence. Such memories would also be less vivid, biased, and intrusive and would include more verbal and fewer sensory elements.

In addition to changes in memories of traumatic events, this study considers improvements in dysfunctional, overly negative appraisals of the trauma and its sequelae [33] as potential mechanisms of change. The theory underlying NET [27] would suggest that symptom reduction in PTSD via exposure methods might also be achieved through reductions in such dysfunctional cognitions related to the trauma. Some evidence already exists of the involvement of dysfunctional trauma-related cognitions in the maintenance of PTSD symptoms in children [34, 35], as well as for changes in trauma-related cognitions predicting PTSD symptom reduction in adults undergoing prolonged exposure [36] and cognitive processing therapy [37].

Fifth, by including a wide range of children without strict exclusion criteria and collecting a wide range of biographical information on these children together with information on peri-traumatic dissociation and cognitive performance, the trial also aims to explore factors possibly limiting the effectiveness of NET. At the same time, it may be possible to identify groups of traumatized children for whom NET is a particularly useful and effective treatment. Including analyses of potential moderators in RCTs has been recommended to reveal possible heterogeneity of effect sizes within samples and to avoid over-generalization of study results [38].

The effects of significant dissociation on the effectiveness of PTSD treatments, especially with children, is still an open question. One trauma intervention among Palestinian children significantly reduced the proportion of clinical posttraumatic stress symptoms only among girls who had a low level of peritraumatic dissociation [39]. On the other hand, for NET in particular, levels of derealization and depersonalization were not found to moderate treatment outcomes with adult refugees in Norway [40]. As regards cognitive performance, Aupperle, Melrose, Stein, and Paulus have presented a model on how cognitive impairments may contribute to the clinical profile of PTSD and lead to the use of alternative coping styles such as avoidance [41]. In light of this possibility, we will also study whether the effects of NET differ according to the children’s level of cognitive performance.

Finally, we study the effects of NET treatment, as compared to a waiting list and TAU, on a number of other outcome variables in addition to PTSD symptom levels. From a practical perspective, the effects of the intervention on depressive symptoms, overall psychological distress as well as resilience and cognitive performance are relevant indicators of clinical effectiveness and impact.

Taking into account the very high levels of comorbid depression in children with PTSD [42], examining whether NET, though targeted at PTSD symptoms, might also reduce depressive symptoms is an important goal. Previous research on prolonged exposure therapy has found that successful treatment of PTSD symptoms lead to reductions in depressive symptoms as well, both in female adults [43] and children [44].

Very few studies have assessed the effects of PTSD treatment on cognitive performance, and there have been calls for more such research [41]. For that reason we also explore possible deficits in attention, working memory, executive functioning and general cognitive performance, their relationship to levels of PTSD symptoms and traumatic exposure, as well as the effects of NET on such deficits, in a subsample of participants. Reviews suggest that in adults some cognitive impairment, at least impaired executive functioning and attention, is associated with PTSD symptoms, as separated from the effects of exposure to trauma per se [45, 46]. However, twin studies of combat-exposed adults suggest this may be at least partly due to higher pre-trauma cognitive capacity acting as a protective factor promoting resilience in the face of traumatic experiences [47].

### Research questions and hypotheses

This study will address the effectiveness of NET in treating trauma-related symptoms and improving resilience and cognitive performance. The mediating roles of trauma-related cognitive appraisals and memory functions on this effectiveness will also be studied, as well as several moderators potentially affecting recovery. Accordingly, the research questions are as follows.

1. Does NET reduce a) posttraumatic stress disorder symptoms, b) depressive symptoms and/or c) psychological distress symptoms in children traumatized by war more effectively than a waiting list condition or treatment as usual? Does NET increase resilience or improve cognitive performance?
  The hypothesis is that NET is more effective in reducing PTSD, depressive and psychological distress symptoms as well as in increasing resilience and improving cognitive performance than TAU or a waiting list condition, to a statistically and clinically significant degree.
2. What changes in the participants’ trauma-related memory functions and cognitions can be observed as a result of NET treatment, as compared to a waiting list condition and treatment as usual?
  The hypotheses are that statistically significant differences between the NET group and the control groups will be found in the participants’ trauma-related memory functions and cognitions at the end of treatment. It is hypothesized that at the end of treatment, NET participants will show lower levels of overgeneralization and fragmentation in their autobiographical memories, as compared to baseline, as well as more spatial and temporal contextualization and coherence. The trauma memories of children after NET are expected to be less biased and intrusive as compared to controls, and include more verbal and fewer sensory elements. Finally, it is hypothesized that participants in the NET group exhibit greater reductions in dysfunctional trauma-related cognitive appraisals, as compared to the waiting list and TAU groups.
3. To what extent do these changes in trauma-related memory functions and cognitions mediate the effectiveness of NET in reducing PTSD symptoms, depressive symptoms and psychological distress symptoms, increasing resilience and improving cognitive performance?
  The hypothesis is that beneficial changes in autobiographical memory, trauma-related memories and trauma-related cognitive appraisals partly explain the reduction in at least PTSD and depressive symptoms among children who participate in NET, and may contribute to increased resilience and improved cognitive performance.
4. To what extent does level of peritraumatic dissociation, level of cognitive performance or gender moderate the effectiveness of NET in reducing trauma-related symptoms?
  The hypothesis is that participants with very high levels of peritraumatic dissociation benefit less from the intervention in terms of symptom reduction. Girls and children with higher levels of cognitive performance are hypothesized to benefit more from the intervention.

## Methods/Design

### Trial design

We are conducting a multicenter, parallel-group, randomized, controlled trial comparing NET to a waiting list condition and TAU at several treatment units located in Finland. Five sets of assessments will be carried out: baseline, T0 (3 months before the start of treatment); pre-test, T1 (when treatment begins); during treatment, T2 (approximately halfway through the treatment); post-test, T3; and follow-up, T4 (three months after treatment). A three-month follow-up period is considered long enough to assess medium-term maintenance of treatment gains, but still short enough to be practically implementable in this context, where drop-outs due to, e.g., asylum seekers being denied asylum and leaving the country, are possible. A three-month follow-up period also means that the T0, T1, T3 and T4 measurements are conducted at equal intervals of three months. Table 1 presents the measurements taken at each point of assessment.

**Table 1:** Points of assessment and measures used

T0 Baseline	T1 Pre-test	T2 Midpoint	T3 Post-test	T4 Follow-up
Approx. three months before start of treatment	At start of treatment	Approx. halfway through treatment	Immediately after treatment	Three months after treatment
– CRIES	– CRIES	– CRIES	– CRIES	– CRIES
– DSRS	– DSRS	– C-PTCI	– DSRS	– DSRS
– SDQ	– SDQ	– TMQQ	– SDQ	– SDQ
– C-PTCI	– C-PTCI	– SUDS (each session)	– C-PTCI	– C-PTCI
– CYRM	– CYRM		– TMQQ	– TMQQ
– TMQQ	– TMQQ		– CYRM	– CYRM
– PDEQ			– CSRS	– CSRS
For subsample:				For subsample:
– WMTB-C				– WMTB-C
– RPM				– RPM
– TMT				– TMT

*C-PTCI*, Children’s Post-Traumatic Cognitions Inventory; *CRIES*, Children’s Revised Impact of Event Scale; *CSRS*, Child Session Rating Scale; *CYRM*, Child and Youth Resilience Measure; *DSRS*, Depression Self-Rating Scale for Children; *PDEQ*, Peritraumatic Dissociative Experiences Questionnaire; *RPM*, Raven’s Progressive Matrices; *SDQ*, Strengths and Difficulties Questionnaire; *SUDS*, Subjective Units of Distress Scale; *TMT*, Trail Making Test; *TMQQ*, Trauma Memory Quality Questionnaire; *WMTB-C*, Working Memory Test Battery for Children

There are two control conditions. First, the time each child spends waiting for treatment to begin, typically three months, will be considered a waiting list control condition. Second, each participant will be randomized into one of two groups. One group will receive NET, acting as the study group, and the other group, receiving TAU, will act as a positive-control group. The trial is parallel-group in nature, with a randomized block design with varying block sizes and an allocation ratio of 1:1.

### Study locations

Most of the interventions will be carried out at three co-operating outpatient clinics and two inpatient wards, all places where traumatized children of immigrant background would in any case receive treatment. These clinics and wards are 1) the Family Counselling Centre of the City of Tampere, 2) the Psychiatric Clinic for Traumatized Children at Tampere University Hospital, 3) the Adolescent Psychiatry Clinic at Tampere University Hospital, 4) the Psychiatric Treatment and Research Unit for Adolescent Intensive Care (EVA) at Tampere University Hospital, and 5) the Centre for Torture Survivors (Helsinki Deaconess Institute). In addition, some participants may be recruited by and receive treatment from co-operating individual therapists operating privately.

### Planned interventions

**Fig. 1:**
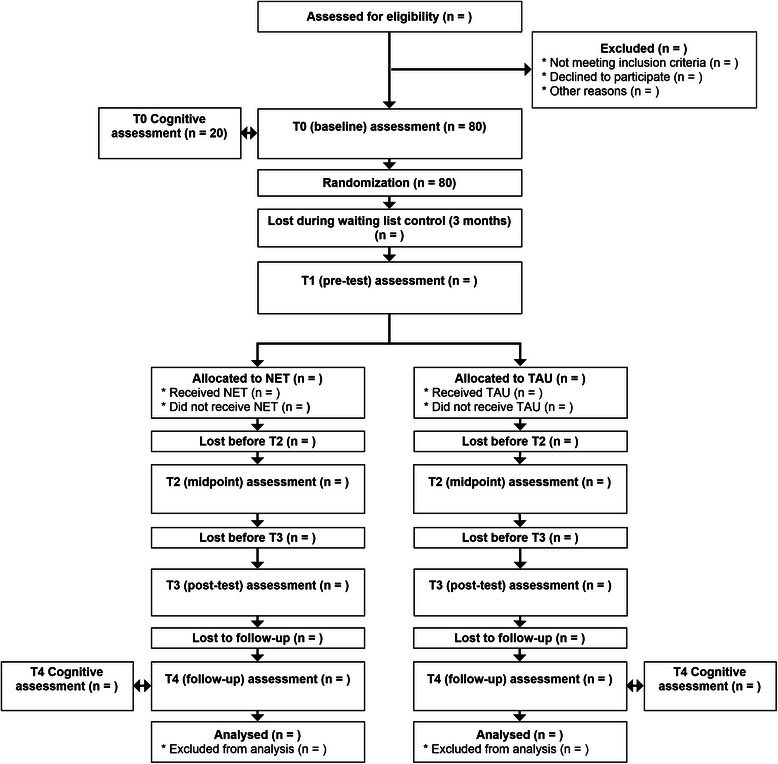
Adapted CONSORT flow diagram. Adapted CONSORT flow diagram, illustrating the study design, the flow of participants in the study and the planned assessments

Figure 1 shows the flow diagram of experimental vs. control situations in the study design.

#### NET intervention

For the intervention group receiving Narrative Exposure Therapy treatment, the intervention will last for approximately three months and include 10–12 weekly sessions of 60–90 min. The course of the intervention will follow the NET manual [14].

The NET intervention begins with a clinical assessment (partly already collected during the T0 measurement), collecting demographic data, and assessing current complaints. This pre-treatment diagnostic session is followed by a session of psychoeducation, which includes explaining the child’s current condition to him/her in a way that helps her/him understand the diagnosis and the purpose of the treatment. After diagnosis and psychoeducation, the narrative exposure portion of the intervention begins. During the first narrative session, the child and the therapist construct a lifeline for the child, with a rope/string representing his/her life from birth up to the present. Next, flowers and stones are placed on the lifeline at chronologically corresponding locations to represent happy moments in life and difficult, fearful or painful moments, respectively. Other symbolic elements may also be used for other significant life events.

The following sessions are generally each devoted to processing a single traumatic event through narrative exposure. Thus, the number of narrative sessions depends on the number of events identified on the lifeline, but should generally be limited to 6–9 weekly sessions. The chronology of the narration during the intervention follows the lifeline, addressing all traumatic incidents throughout the course of the child’s biography, accompanied by other turning points in her/his life. In each session, the therapist writes down the child’s narration. In the subsequent session, the narrative from the previous session is read aloud to the child, and the child is asked to correct it or add any details that may have been missed. The procedure is repeated in subsequent sessions until a final version of the child’s biography is created. In the last session, the biography as a whole is read aloud to the child. The child and the therapist sign the written narration. One copy is handed to the child. Another is kept for research purposes, provided that permission for this is obtained.

All clinicians providing treatment will receive a three-day training on NET by instructors experienced in the method. Each participating unit will be provided with NET manuals. For the NET intervention group, clinicians are instructed to adhere as strictly as possible to the intervention as laid out in the NET manual. Semi-annual supervisory meetings will be organized with the clinicians and the research team to discuss the use of the treatment method and any issues in implementing it that may have appeared. The developers of the method are also available for supervisory purposes via Skype.

#### Control intervention

For the TAU control group, participants will receive the usual care for posttraumatic stress symptoms currently offered by each unit. This treatment may last for more than the three months the NET intervention will take, but T3 measurements will in any case be collected three months after treatment has started. No specific instructions will be given to clinicians as to what TAU should entail, apart from not including elements specific to NET. Thus, in keeping with the pragmatic goal of studying the effectiveness of NET as compared to current usual care, the TAU provided by clinicians at cooperating units will vary and include whatever methods they have used until now. These methods are likely to include the use of (TF-)CBT, Eye Movement Desensitization and Reprocessing and forms of family therapy. Information about the kind of treatment offered to each participant in the control group will be collected.

### Participants

The participants of the study are up to 80 children (between 9 and 17 years of age) living in Finland who will attend Narrative Exposure Therapy or treatment as usual for posttraumatic stress symptoms due to exposure to armed conflict. As no war or major organized violence has taken place in Finland for decades, these participants will be immigrants, refugees or asylum seekers. The sample will be collected among all eligible children who are clients of (a) The Family Counselling Center of the City of Tampere, (b) The Psychiatric Clinic for Traumatized Children at Tampere University Hospital, c) The Adolescent Psychiatry Clinic at Tampere University Hospital, d) the Psychiatric Treatment and Research Unit for Adolescent Intensive Care at Tampere University Hospital, e) The Centre for Torture Survivors of the Helsinki Deaconess Institute or f) co-operating individual private practitioners trained in NET.

#### Inclusion and exclusion criteria

Inclusion and exclusion criteria applied to clinical research on PTSD vary widely, with no established standards [48]. In keeping with the aim of carrying out a pragmatic trial in usual care settings, this trial aims for a great variety of participants with few exclusion criteria. Thus, only acute psychosis or marked intellectual disability will be considered definite criteria for exclusion, while other comorbid conditions such as depression, generalized anxiety disorder or obsessive-compulsive disorder will not. Generally, any child between 9–17 years old who a) has spent some part of her/his life living in a country where organized violence was taking place, or at a refugee camp, and b) suffers from significant stress symptoms thought be trauma-related, may be included in the study. All children will be clinically assessed at the start of treatment, but a formal diagnosis of PTSD is not required.

### Measures

#### Primary outcomes

*Posttraumatic stress symptoms* will be measured at T0, T1, T2, T3, and T4, employing the children’s version of the Revised Impact of Event Scale (CRIES) [49]. CRIES consists of 13 items covering the re-experiencing, avoidance and hyperarousal symptom dimensions. Children estimate the occurrence of each symptom on a 4-point scale. CRIES-R has been found to have good reliability among war-affected children [50].

*Depressive symptoms* will be measured at T0, T1, T3, and T4 employing the Depression Self-Rating Scale for Children (DSRS) [51]. The measure includes 18 items that assess the cognitive, affective, and behavioral dimensions of depression. At each item, children estimate on a 3-point scale whether they have experienced the symptom over the preceding two weeks.

*Psychological distress* will be measured at T0, T1, T3, and T4 employing the Strengths and Difficulties Questionnaire (SDQ) [52]. One form is provided for self-assessment by the child and another for a parent or guardian, if available. The measure covers emotional and behavioral problems and hyperactivity. Each dimension consists of 5 items evaluated on a 3-point scale as to how well the description fits the situation of the child.

*Resilience* will be measured at T0, T1, T3, and T4 using an adapted, focus-group-based version of the Child and Youth Resilience Measure (CYRM) (28-item version originally developed by Ungar and Liebenberg [53], and validated in [54]). CYRM is a questionnaire exploring the individual, relational, communal and cultural resources that may bolster the resilience of 9–23-year-old youth. The participant reports on a 5-point scale as to what extent he/she feels he/she has certain resources.

*Cognitive performance (of a subset of participants)* will be addressed by measuring a) attention and working memory, b) non-verbal, general cognitive performance and c) executive functioning. Attention and working memory will be measured at T0 and T4 employing performance-based sub-tests for verbal and spatial working memory from the Working Memory Test Battery for Children (WMTB-C) [55]. Non-verbal, general cognitive performance will be assessed at T0 and T4 employing age-appropriate versions of Raven’s Progressive Matrices (RPM) [56]. Executive functioning will be measured at T0 and T4 employing the Trail Making Test (TMT) [57].

#### Secondary outcomes and mediators

*Quality of traumatic memory* will be assessed at T0, T1, T2, T3, and T4 employing the Trauma Memory Quality Questionnaire (TMQQ) [58]. The measure contains 11 items related to the visual quality, non-visual sensory qualities (e.g., auditory, olfactory and proprioceptive sensations), and temporal context of the traumatic memory, as well as to the extent to which the memory is in a verbally accessible format. Participants evaluate each item on a 4-point scale as to how much they agree with the statements.

*Trauma-related cognitions* are measured at T0, T1, T2, T3, and T4 using the Children’s Post Traumatic Cognitions Inventory (C-PTCI) [59]. The measure includes 25 statements on the kinds of thoughts and feelings the child has had after the traumatic event, e.g., “I feel like I am a different person since the frightening event.” Children evaluate on a 3-point scale how well the descriptions fit them.

*General functioning of autobiographical memory* will be explored, in the intervention group only, by tracking the number of life events initially identified at the start of the intervention, as well as the recovery of new events and possible temporal reorganization of life events during therapy. In addition, the written life narratives that result from NET treatment will be coded for (1) emotional tone (positive vs. negative), (2) thematic lines (agency and communion), and (3) narrative complexity [60], as well as for sequences of redemption and contamination [61].

#### Additional measures: moderators and measures related to therapeutic interaction

*Peritraumatic dissociation* will be assessed at T0 using the Peritraumatic Dissociative Experiences Questionnaire (PDEQ) [62]. The measure assesses the level of dissociation at the time of the traumatic event and immediately after it. 10 items are evaluated on a scale from 1 (not true at all) to 5 (extremely true).

*Cognitive performance* at T0, measured as described above, will also be treated as a possible moderator.

*Distress during the therapeutic intervention* is measured several times during each treatment session using the Subjective Units of Distress Scale (SUDS) [63]. The level of distress is measured in order to guarantee the safety of the intervention as well as to obtain data on the intrusiveness of narratives. SUDS consists of a thermometer-like scale from 0 to 10 that measures the subjective intensity of disturbance or distress currently experienced by the participant. When prompted, the child points to or otherwise indicates where he/she is at on the scale at the moment. SUDS is commonly used as a benchmark for a professional or observer to evaluate the progress of treatment.

*Intervention effectiveness and effective treatment components from the client’s point of view* are assessed with the Child Session Rating Scale (CSRS) [64], administered at T3. In the CSRS, the child evaluates five dimensions of interaction during the intervention by marking a point on a line segment.

### Procedure

A sequential procedure will be applied to data collection. Whenever a child is identified as a potential participant by a clinician at one of the co-operating units, information concerning the intervention and the related research is provided both to the participant him/herself and to his/her parents. If they are willing to participate in the research, informed consent will be requested from the child and his/her parents.

Waiting list control baseline data (T0) will be collected during this first meeting with the clinician. The children will then be randomly assigned to EITHER the ‘treatment as usual’ group (n = 40) OR the ‘NET intervention’ group (n = 40). Because of the treatment queues currently present at most of the treatment units, all participants will first be placed under waiting-list conditions for approximately three months (waiting list control condition, n = 80). In the unlikely case that there is no treatment queue at the moment, the participant will begin NET or TAU immediately and will not contribute waiting list control data.

At the start of the intervention or TAU, pre-test (T1) data will be collected from all participants. The T1 data will serve as an end measurement for the waiting list control condition and as the pre-test measurement for the NET intervention and the TAU control condition.

Data (T2) will also be collected once during the treatment for both the NET and the TAU groups. This will allow for proper analysis of mediating mechanisms. In the NET group, this assessment will take place at the time when the worst traumatic memories have been narrated.

Post-test data (T3) will be collected from participants in both groups, immediately after the intervention for the NET group, and after three months of treatment for the TAU group, and follow-up data (T4) will be collected during a joint family meeting, three months later.

In most cases, the clinician working as the therapist will also act as the assessor and collect data from the patients he/she is treating. In some cases, members of the research team may carry out some assessments, particularly for follow-up. Interpreters are used whenever needed during assessments, and will be present during all assessment and treatment sessions for children who are not fluent in Finnish.

The procedure will be continued until the sample size reaches the goal of n = 80, with data collection estimated to finish during 2017.

Due to restrictions on the use of the tests used for measurements of cognitive performance, these additional assessments will be carried out by a member of the research team (SK), a licensed clinical psychologist, and studied in a smaller subsample. For n = 20 participating children about to start treatment during 2015, willingness and informed consent to participate in these additional tests will be requested, and the measurements will then be carried out at T0 and T4 for this subsample.

### Sample size calculation

The sample size was calculated using G*Power 3.1.9.2 [65]. Earlier results obtained on NET with children [5, 31] suggest large between-groups effect sizes of *d =* 0.7–1.0 for PTSD symptom severity for NET vs. waiting list. For determining the effectiveness of NET vs. waiting list, with α = 0.05, *d =* 0.85 (*f ≈ 0.40)* and a priori test power = 0.80 (β = 0.20), 52 participants would be required for analyses of covariance of fixed effects, main effects and interactions on primary outcomes.

There are no effect size estimations available for TAU as offered to traumatized children at participating clinics, nor could such estimations be reliably made, taking into account the diverse nature of TAU expected. However, at least some effectiveness for TAU over a waiting list can be assumed. Thus, in order to tease out differences in NET vs. TAU between-treatments comparisons, we needed to aim for a sample size that is somewhat larger than that required for detecting NET vs. waiting list between-groups differences. At the same time, dropout rates for participation in NET reported in earlier studies are generally very low [29, 66], so little attrition is expected. In light of these considerations and practical limitations, we aim for a sample of 80 participants.

### Randomization

To continuously provide close to equal numbers of participants in the control and treatment groups for interim analysis, random allocation is performed using a randomized block design with variable block sizes and an allocation ratio of 1:1 [67].

At the start of the study, before any participants were recruited, the participating clinics were provided with folders by the research group, each containing all the relevant research material, questionnaires and measurements for one participant. A sealed envelope was placed by the research team in each folder with a piece of paper inside. Half of these papers were marked “NET” and the other half “TAU”. Whenever a suitable participant is identified at a study location by a clinician and informed consent is received from her/him and her/his parents or guardians, the clinician will randomly select a folder for her/him. The envelope will then be opened and its content will determine whether the participant receives NET or TAU. Allocation was concealed by the use of opaque, sealed envelopes.

Due to most participating clinicians acting both as treatment providers and assessors, blinding them as to the treatment status of each participant beyond randomization (after T0) is not possible and will not be attempted. The participants themselves cannot be blinded to the intervention, as the differences between TAU and NET will make it obvious to them which group they belong to.

### Statistical analyses

#### Basic analyses

The sample and its characteristics will be analysed employing descriptive statistical methods. Means, standard deviations and other basic statistics for all relevant variables will be calculated for descriptive purposes. Between-group comparisons at baseline will be performed to test randomization and assess differences between treatment and control groups, as well as between participants at different study locations. The internal reliabilities of the instruments used will be analysed employing Cronbach’s α. For all statistical tests, significance levels will be set at α = 0.05, with the Holm-Bonferroni method used to control family-wise error rates, where appropriate. Multiple imputation will be employed in the case of dropouts or other missing data. Intention-to-treat analyses will be carried out, so that all participants who are randomized into either group will be included in final analyses.

#### Effect evaluation

The main effect of NET as compared to TAU and the waiting list control condition on the primary outcomes of PTSD symptom severity, depressive symptoms, psychological distress, resilience and cognitive performance will be analysed employing analyses of covariance. Potential confounding factors (age, gender, treating unit) will be included as covariates. The main effects of NET as compared to TAU and the waiting list condition on the secondary outcomes of trauma memory quality and trauma-related cognitions will be similarly analysed employing analyses of covariance. For all analyses of covariance, effect sizes will be reported as partial η^2^, with 95 % confidence intervals included.

#### Moderator and mediator analyses

Analyses of the potential mediating and moderating factors will be carried out employing methods of conditional process analysis, based on [68, 69]. The end result will be presented as a process model of the effects of NET on PTSD and other symptom measures.

### Ethical issues

The Regional Ethics Committee of Tampere University Hospital has approved the study (R14065, 6/2014), deeming it ethically acceptable and fulfilling the requirements of the Finnish Medical Research Act (488/1999) and the corresponding Decree (986/1999).

Age-specific brochures with information about the study have been prepared for the participating children and their guardians, and translated into several languages. Written consent will be requested from both parents or guardians and the children themselves.

The anonymity of the subjects will be guaranteed by removing identity information when analyzing the data. All data with names and identity information will be destroyed after a period of five years. The data protection ombudsman has been notified of the data collection procedures and the formed register of personal data in line with the Finnish Personal Data Act (523/1999), §10 and §14. The trial has been registered as clinical trial number NCT02425280 in the ClinicalTrials.gov Protocol Registration System on 15 April 2015.

Adherence to the treatment paradigm and procedures will be guaranteed by instructing clinicians to adhere to the NET manual as strictly as possible and by organizing semiannual supervisory meetings.

Interim analyses of differences in treatment outcomes between NET and TAU will be carried out after all measurements are available from the first 20 participants. If, at this time, NET treatment is found to be performing worse than TAU to a clinically significant extent, the trial will be stopped due to ethical concerns.

## Discussion

The practical need for trials such as this is evident and pressing. Millions of children around the world suffer from posttraumatic stress symptoms due to experiences of war and organized violence, and providing them with the most effective forms of evidence-based help is a crucial, global concern. As of yet, we still have limited evidence, especially from RCTs, on the effectiveness of interventions targeted at war-affected children. A standardized, manualized intervention like NET with wide applicability has the potential to become a very useful tool for providing help to such children, both in their original home countries and as immigrants, refugees or asylum seekers in other countries. Thus, providing evidence for its effectiveness in a variety of contexts makes for clinical research of great practical importance.

At the same time, even as CBT-based interventions seem to be effective in reducing PTSD symptomatology, we have no comprehensive view based on theory as to exactly how and in what conditions this happens. As such, the results of this trial may contribute significantly to our understanding of the mechanisms of change that are crucial to successful treatment of PTSD in children. The theory underlying NET suggests that improved contextualization of traumatic memories is central to recovery from PTSD, and thus we expect that improvements in the quality of traumatic memories will mediate its effectiveness. Results on the possible mediating role of decreased dysfunctional trauma-related cognitive appraisals in treatment effectiveness, as suggested by recent research [36,37], further contribute to the search for active mechanisms of change. Exploring the possible effects of treatment on elements of cognitive functioning will shed light on the relationships between level of cognitive performance, trauma and PTSD. Although a great variety of findings on cognitive impairment in PTSD exist, there is still significant disagreement as to which of these findings represent pre-trauma susceptibility and resiliency factors or effects of comorbid conditions and which toxic effects of trauma or PTSD [45-47].

Further, including exploration of the potential moderating effects of peri-traumatic dissociation, gender and level of cognitive performance on treatment effectiveness in this trial adds to its potential to help guide the future development of intervention methods.

### Generalizability of study results

As a pragmatic trial studying real-world effectiveness in a usual care setting, the contributions of this trial to assessing the practical usefulness and applicability of NET in Finland are direct and immediate. As effectiveness is studied directly in the healthcare system itself, there will be no need for a separate implementation and roll-out phase, should NET be found effective and applicable in this context. In addition, as no extra resources as such will be provided for participating units, successfully carrying out NET interventions during the study will be proof of the feasibility of using NET with current resource and time constraints in these units.

The results of this study will be generalizable to healthcare settings in the Nordic countries and most other high income countries where therapeutic interventions such as NET may be provided to immigrant children. The results will be less generalizable to care environments where healthcare services are organized in a very dissimilar manner or where significantly less resources are available for provision of healthcare.

### Risks and limitations

Though it is possible for participating children to temporarily experience increased stress and discomfort during NET sessions, long-term iatrogenic risks are not expected in this trial. However, interim analyses will be carried out to ensure NET is not performing worse than TAU to a clinically significant extent.

One notable challenge in this trial will be the question of language. The majority of therapeutic work carried out in this trial will likely take place with the help of interpreters. Whenever possible, the same interpreter will work with the same child in all NET or TAU sessions, and interpreters will be given instructions to work as literally as possible. Constant cooperation with the organization providing interpreters will be maintained throughout the study to ensure recruitment of interpreters who are familiar with trauma and therapeutic methods in general, as well as with the special requirements of the NET method. Results from earlier NET studies seem to suggest no significant differences in outcomes between using and not using interpreters [29]. However, especially as we are dealing with a method placing great emphasis on narration and verbalization, the use of interpreters may pose a variety of practical problems and must be considered a possible source of confounding effects on study results.

Another challenge for the trial is its reliance on mostly child-evaluated measures. For many of the factors under study, other forms of reliable measures do not exist. In addition, as clinicians will act as assessors for the most part in this trial, the battery of measures and instruments has to be kept relatively simple and manageable in scope. In any case, for some measures, such as the qualities of traumatic memories, it can be expected that self-evaluations by, in particular, the youngest children taking part will also present potential sources of confounding effects.

The varied nature of the treatment-as-usual condition we are comparing NET to also poses challenges. On one hand, we have attempted to mitigate this problem by also including a waiting list condition, and by collecting information about the kind of TAU offered to each child. On the other hand, this variety in TAU is to be expected in a pragmatic framework and is a realistic reflection of the varied practices currently in use at the participating treatment units.

## Conclusions

In summary, this article has described the rationale and design of a randomized controlled trial studying the effectiveness and prospective mechanisms of change of Narrative Exposure Therapy in the treatment of posttraumatic stress symptoms in children affected by war or armed conflict. Overall, the results of this trial will contribute to basic research on PTSD, memory processes and cognitive functions in children, as well as to applied research into therapeutic interventions and their development. Beyond the specific analyses described in this article, the data collected will be utilized in further interdisciplinary collaboration with researchers from the fields of political geography and social anthropology. In this context, qualitative analysis of the life stories resulting from NET is under consideration. We also hope to utilize the results of this study in future international comparative research, e.g., by exploring the effects of treatment location (country of origin vs. new home country) on the effectiveness of NET.

## Trial status

The trial started in December 2014 and data collection is expected to continue until 2017, with continuous participant enrolment.

## Abbreviations

C-PTCI: Children’s Post-Traumatic Cognitions Inventory
CBT: Cognitive-Behavioral Therapy
CRIES: Children’s Revised Impact of Event Scale
CSRS: Child Session Rating Scale
CYRM: Child and Youth Resilience Measure
DSRS: Depression Self-Rating Scale for Children
NET: Narrative Exposure Therapy
PDEQ: Peritraumatic Dissociative Experiences Questionnaire
PTSD: Posttraumatic Stress Disorder
RCT: Randomized Controlled Trial
RPM: Raven’s Progressive Matrices
SDQ: Strengths and Difficulties Questionnaire
SUDS: Subjective Units of Distress Scale
TAU: Treatment as usual
TF-CBT: Trauma-Focused Cognitive-Behavioral Therapy
TMT: Trail Making Test
TMQQ: Trauma Memory Quality Questionnaire
WMTB-C: Working Memory Test Battery for Children
